# Prognostic Value of the Residual SYNTAX Score on In-Hospital and Follow-Up Clinical Outcomes in ST Elevation Myocardial Infarction Patients Undergoing Percutaneous Coronary Interventions

**DOI:** 10.1155/2020/9245431

**Published:** 2020-10-29

**Authors:** Refik Emre Altekin, Ali Yasar Kilinc, Mehdi Onac, Orhan Cicekcibasi

**Affiliations:** ^1^Akdeniz University, Faculty of Medicine, Department of Cardiology, Antalya, Turkey; ^2^Akcakoca State Hospital, Department of Cardiology, Akcakoca, Duzce, Turkey

## Abstract

**Aims:**

We investigated the prognostic significance of residual SYNTAX score (RSS) in patients undergoing PCI due to STEMI and relationship between RSS and in-hospital and long-term ischemic cardiac events.

**Methods:**

Between June 2015 and December 2018, 538 patients who underwent primary PCI were evaluated for in-hospital events and 478 patients were evaluated for clinical events during follow-up. Primary and secondary endpoints for both in-hospital and follow-up periods were cardiac death and major adverse cardiac events (MACE).

**Results:**

538 patients were included the study. RSS values of 131 patients were 0, and RSS values of 407 patients were >0. The median value of the RSS > 0 group was 7. According to this value, the RSS > 0 group was divided into 2 groups as R-ICR (RSS < 7, *N* = 188) and ICR (RSS ≥ 7, *n* = 219). In the RSS ≥ 7 group, during in-hospital and follow-up period, both mortality and MACE rates were higher than the other two groups. Area under the curve (AUC) for RSS for in-hospital death was found to be higher than SS (*p*=0.035) but similar to Grace Score (GS) (*p*=0.651). For MACE, RSS was higher than SS (*p*=0.025) and higher than the GS (*p*=0.041). For follow-up cardiac mortality, the AUC of the RSS was found to be higher than SS (0.870/0.763, *p*=0.02) and GS (0.870/0.733, *p*=0.001). For MACE, the AUC of RSS was higher than SS (*p*=0.03) and GS (*p*=0.004).

**Conclusions:**

High RSS values in STEMI patients are associated with increased risk of ischemic cardiac events. RSS may help determine revascularization and level of additional PCI to improve prognosis by reducing the risk of ischemic cardiac events after P-PCI.

## 1. Introduction

ST-elevation myocardial infarction (STEMI) is known as a life-threatening complication of coronary artery disease (CAD) and it is one of the leading causes of death all over the world. Primary percutaneous coronary intervention (P-PCI) of the culprit vessel in patients with STEMI is standard clinical practice [1]. At the time of P-PCI, 4065% of the patients exhibit one or more concomitant coronary lesions (i.e., multivessel disease (MVD)). The presence of narrowed coronaries other than those related to index ischemia in patients with STEMI is suggested as a feature associated with adverse clinical outcomes [2]. The presence of MVD is strongly associated with higher 30-day mortality, reinfarction ratio, reduced myocardial reperfusion success, and occurrence of major adverse cardiac events (MACE) at 1 year compared with single-vessel CAD [3, 4].

Complete revascularization (CR) of all diseased segments is a desirable goal in patients undergoing P-PCI. Despite major advances in PCI technology and technique, CR is often not achieved (e.g., the diffuse disease in small vessels, complex calcified lesions, and chronic total occlusions). Also, CR has always been an important topic of discussion. Indeed, incomplete revascularization (ICR) may be unavoidable and detrimental in patients with complex coronary artery disease [5–8]. In cases where CR is not possible, PCI can be made to ensure a minimum level of ischemia to prevent ischemic cardiac events. The concept of reasonable ICR, defined as a reasonable amount of coronary lesion burden after revascularization, is on the agenda because it gives similar results with patients who underwent CR. The effect of residual SYNTAX score (RSS) for the determination of a reasonable ICR level was investigated in patients with MVD who underwent PCI [5, 9, 10].

The RSS was developed to quantitatively assess the degree and complexity of residual stenoses, based on recalculating the SYNTAX score from coronary angiography after PCI [11]. Systematic characterization and quantification of residual atherosclerosis after PCI may be important to standardize and improve the prognostic utility of ICR. Higher RSS has been associated with worse outcome in patients undergoing angiography-mediated PCI [12]. In a post hoc analysis of the ACUITY trial including patients with moderate-high-risk non-ST elevation (NON-STEMI) acute coronary syndromes, RSS > 8.0 was an independent predictor of mortality and ischemic events at one year [13].

In this study, we investigated the prognostic significance of RSS in patients undergoing PCI procedures due to STEMI and its relationship with ischemic cardiac events during hospitalization and follow-up period.

## 2. Materials and Methods

**Figure 1:**
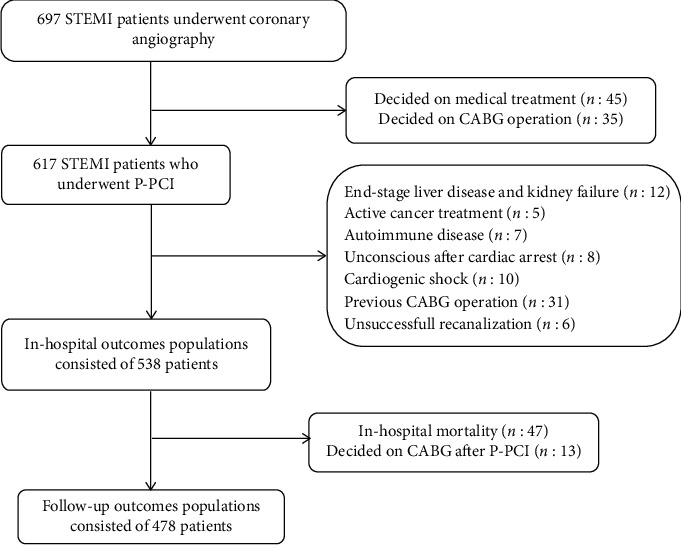
Flow diagram demonstrating enrollment and follow-up study patients.

This was a single-center, retrospective, observational cohort study that enrolled consecutive unselected nonrandomized eligible patients who were hospitalized for STEMI and underwent P-PCI at our institution from June 2015 to December 2018. To determine adequate sample size, G ∗ Power 3.1.9 was used [14]. Effect size was determined as *w* = 0.291. With the 95% power and 0.05 alpha, the minimum sample size to be included in the study was calculated as *n* = 183. A total of 697 patients were screened for involvement in study. 159 patients were excluded from the study. After angiography, 45 patients were given medical treatment, and 35 patients were given CABG decisions. In addition, patients with severe concomitant noncardiac diseases were excluded from the study. This is shown in Figure 1. A total of 538 patients were included to the final study population. Clinical events of patients, which developed in-hospital and after discharge until June 2019, were recorded and retrospective analysis was performed. Ischemic cardiac events that developed in-hospital and after discharge were evaluated separately. Our local ethics committee approved the study protocol by the Declaration of Helsinki and all patients provided written informed consent.

Demographic, clinical, and laboratory data at admission were collected from our hospital electronic database. Patients who were previously diagnosed with diabetes or newly diagnosed according to ESC Diabetes Guideline were classified as diabetic [15]. Patients who were previously diagnosed with hypertension and those with values above 140/90 mm·Hg in serial blood pressure measurements after STEMI were categorized as hypertensive. Dyslipidemia was defined as a low-density lipoprotein cholesterol level of 130 mg/dl or more at fasting [16]. However, patients who were previously diagnosed with dyslipidemia or who used lipid-modifying agents were also included in this category. Patients who smoked within 12 months before the index event were classified as smokers. Patients who had undergone PCI before and who had ≥50% stenosis in major coronary arteries and side branches, larger than 1.5 mm, on coronary angiography were defined as having coronary artery disease. The diagnosis of STEMI was made according to current guidelines [17].

In our study, biochemical and hemogram data were obtained from blood samples taken at admission. Cardiac enzymes, creatinine levels, and hemogram parameters were studied daily during hospitalization. Peak troponin levels during hospitalization were evaluated in the study. Since troponin values above 50000 are given as >50000 in laboratory results, this situation was taken into consideration in our study. Contrast-induced nephropathy (CIN) was defined as a 25% increase in creatinine level compared to baseline or as an absolute increase of at least 0.5 mg/dl in the first 48 hours after P-PCI [18]. Major bleeding was defined according to the Bleeding Academic Research Consortium (BARC) definition (BARC type 3–5) [19]. Grace Score (GS) was calculated by using clinical, laboratory, and electrocardiographic data at admission [20]. All patients underwent echocardiography within 48–72 hours after P-PCI and after discharge at the first month. The modified Simpson method was used to calculate the left ventricular ejection fraction (LVEF).

### 2.1. Invasive Procedures

P-PCI was undertaken according to the European Society of Cardiology Guidelines and the operator's routine practice. In the centers where the patients are referred or in our emergency department since clopidogrel or ticagrelor was given as a P_2_Y_12_ inhibitor, the rate of use of these drugs was high. The exchange between the molecules of interest was left to the operator's preference. In patients who did not receive clopidogrel and ticagrelor before coronary angiography, prasugrel was also given before P-PCI depending on operator preference. The selection of the specific type of revascularization, procedural devices, and stent types was based on the decision of the operator. After the successful procedure, in line with the current STEMI guidelines, the patient's clinical characteristics were taken into consideration and their treatments included aspirin, P_2_Y_12_ inhibitor, beta-blocker, statin, angiotensin-converting enzyme inhibitor-angiotensin receptor blocker, mineralocorticoid receptor antagonists, and furosemide [17].

The coronary arteries were divided based on anatomical criteria. The following vessels were considered as major coronary arteries: left main coronary artery (LMCA), left anterior descending (LAD), circumflex (CX), and right coronary artery (RCA). Isolated intermediate and isolated diagonal artery lesions were classified as other coronary vessels (if there is no critical stenosis in the three major coronary arteries). MVD was defined as at least one lesion in a major non-infarct-related artery deemed angiographically significant (≥50% luminal narrowing diameter). Ischemia time was defined as the time between symptom onset and reperfusion (after balloon angioplasty or direct stent implantation in the culprit artery during P-PCI). Thrombolysis in myocardial infarction (TIMI) flow grade at the start and end of the procedure was determined from the angiographic films as previously described in previous studies [21]. Stent diameter refers to the maximal stent diameter, and stent length is the sum of the lengths of all implanted stents. Multivessel PCI was defined as revascularization of at least one non-culprit lesion during the index procedure, before discharge or planned in the following 30–45 days without ischemic symptoms. Treatment of non-culprit artery during P-PCI was left to the operator's preference. The decision to revascularize the non-culprit coronary artery after P-PCI was made by the heart team.

### 2.2. Baseline and Residual SYNTAX Score

Coronary angiograms were recorded to digital media for quantitative analysis. The SS and RSS were derived from the summation of the individual scores for each lesion (defined as ≥50% stenosis in vessel ≥1.5 mm) on angiograms obtained before and after the procedure, respectively, as previously described [22]. All angiographic variables pertinent to SS and RSS calculations were computed by two of the experienced cardiologists who trained for SS assessment and they were blinded to procedural data and clinical outcomes. In case of disagreement, the opinion of the third observer was obtained and the final decision was made by consensus. All data were assessed for quality and entered a dedicated computerized database. In our study, RSS score calculated after the procedure was evaluated in the results of the hospital, which was calculated after the intervention to IRA or after additional coronary vascular interventions outside IRA before discharge. If additional vascular intervention was not planned after discharge, the same RSS value was used for follow-up results. In patients who underwent additional vascular intervention after discharge, RSS value calculated after PCI during the hospital was recorded in the in-hospital results. RSS calculated after additional planned PCI in the follow-up was used for the results in the follow-up period. The author evaluating the result was blind to the SS and RSS score.

### 2.3. Follow-Up, Clinical Endpoint Definitions

We divided endpoints into two groups as in-hospital and follow-up. Information about in-hospital outcome was obtained from an electronic centralized clinical database. In-hospital clinical results were collected by a physician who was unaware of the initial clinical, laboratory, and angiographic results. In-hospital primary endpoint was defined as incidence of cardiac death considered as any death with a demonstrable cardiac cause or any death that was not attributable to a noncardiac cause. In-hospital secondary endpoint was MACE, the combined endpoint of cardiac death, reinfarction, heart failure, and arrhythmic events. Reinfarction in acute post-P-PCI phase was defined as clinical signs of reinfarction with recurrent or persistent symptoms and ST-segment changes and requiring repeat P-PCI and/or second peak in CK-MB mass or troponin-T increase to ≥3 times the upper limit of normal, not related to an interventional procedure and new pathological *Q* waves in 2 or more contiguous electrocardiograph leads [8]. Heart failure was defined as presence of signs or symptoms of congestion, mainly shortness of breath, and signs of fluid retention. Arrhythmic events during hospitalization were defined as documented sustained ventricular tachycardia (VT) and ventricular fibrillation (VF). Safety endpoints were defined as combined endpoints of CIN and bleeding.

After discharge, at follow-up, clinical evaluation was made with 478 patients because 47 patients died in hospital and 13 patients decided to undergo surgical treatment after P-PCI (Figure 1). All patients were contacted for follow-up to assess the presence of primary and seconder endpoints. After discharge, all clinical follow-up data were prospectively collected by scheduled clinic evaluations, in-hospital records of the rehospitalized patients, and direct telephone interviews. In cases of the unavailable information, we obtained information from the local citizen's registration office and medical charts. Follow-up clinical events were investigated and recorded by a physician unaware of RSS score and in-hospital events.

Follow-up primary endpoint is cardiac death and secondary endpoint is MACE, which consists of combined endpoints of cardiac death, non-fatal reinfarction, heart failure, and recurrent revascularization. Cardiac death was defined as death caused by myocardial infarction, heart failure, sudden cardiac death, and cardiac procedures. Nonfatal reinfarction was defined as a new elevation of cardiac biomarkers associated with symptoms or new *Q* waves at 12-lead electrocardiogram or ST-T variation during symptoms [23]. Patients hospitalized with the diagnosis of decompensated heart failure were evaluated as clinical endpoint of heart failure. Any repeat revascularization was defined as any ischemia-driven target or non-target vessel revascularization by either PCI or CABG [24].

### 2.4. Statistical Methods

Statistical analysis was made by using IBM SPSS Statistics for Windows, Version 23.0 (IBM Corp., Armonk, NY). The normality assumptions were controlled by the Shapiro–Wilk test. Descriptive analyses were presented by using mean ± SD, median (0.250.75 percentiles), or *n* (%), where appropriate. Categorical data were analyzed by Pearson chi-square. The Kruskal–Wallis test was used for comparison of non-parametric variables between groups and the Bonferroni–Dunn test was used as a post hoc test for significant cases while One-Way ANOVA with post hoc Tukey HSD test was used for parametric variables. The receiver operating characteristic (ROC) analysis was applied to evaluate the predictive performance of SS, RSS, and GS for MACE and cardiac death and area under the curve (AUC), sensitivity, and specificity were calculated and reported with 95% confidence intervals. The optimal cutoff point of measurements was determined as the value of the maximum Youden index. Survival curves were generated by the Kaplan–Meier method, and the log-rank test was used to evaluate differences between groups. Univariate and multivariate analyses of independent predictors of cardiac death and MACE were performed with a Cox proportional hazard regression model. The variables which showed significant association with cardiac death and MACE in the univariate analyses were further tested in the multivariate model. Hazard ratio (HR), with corresponding 95% confidence intervals (95% CIs), was reported. A *p* value of less than 0.05 was considered statistically significant.

## 3. Results

RSS values of 131 patients were 0, and RSS values of 407 patients were >0. The median value of the RSS > 0 group was 7. According to this value, RSS > 0 group was divided into 2 groups as R-ICR (RSS < 7, *N* = 188) and ICR (RSS ≥ 7, *n* = 219). Finally, patients were divided into 3 groups as RSS = 0, RSS < 7, and RSS ≥ 7 according to mean RSS value. In our study, the mean follow-up duration was 29 (18–35) months, and there was no difference between the groups in terms of follow-up duration.

**Figure 2:**
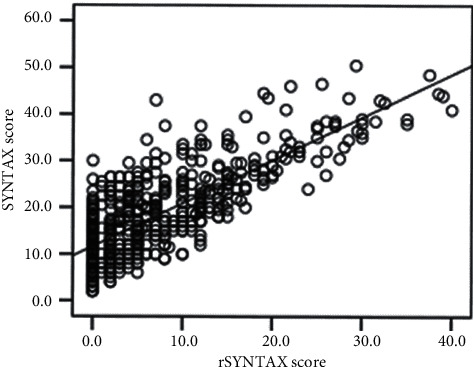
Correlation histogram between basal SS and RSS (*r* = 0.727; *p* < 0.001) Spearman test.

Baseline demographic, clinical, and laboratory data were analyzed and compared between the groups (Table 1). Patients with RSS ≥ 7 were older and had a higher prevalence of CAD. In the RSS ≥ 7 group, the number of admissions with Killip ≥2 heart failure, need for intravenous diuretic, inotropic therapy, and intra-aortic balloon pump was higher than the other groups; moreover, duration of hospitalization was longer in this group. Blood glucose, HbA1C, creatinine, leukocyte, CRP levels, and GS were higher in the RSS ≥ 7 group, and systolic and diastolic blood pressure, creatinine clearance, and LVEF were lower than the other groups. The ratio of hypertensive, diabetic patients and neutrophil levels in the RSS ≥ 7 group was higher than the RSS = 0 group. Peak troponin levels and ischemia time were significantly different between all groups. There was a positive correlation between basal SS values and RSS values of the patients included in the study (*r* = 0.727; *p* < 0.001). We show this correlation in Figure 2.

The angiographic and procedural characteristics of the patients included the study are presented in Table 2. In the RSS ≥ 7 group, pre-PCI TIMI-0 flow, and post-PCI TIMI-2 flow rates were higher than the other groups. Post-PCI TIMI-3 flow, the rate of infarct-related artery as LAD, and additional vessel intervention rates were low. Post-PCI TIMI-1 flow and infarct related artery (IRA) RCA ratios were higher in RSS ≥ 7 group than RSS = 0 group. The rate of two-vessel disease was higher in the RSS < 7 groups than the other groups. Single-vessel, three-vessel, and multivessel disease rates and SS and RSS levels were significantly different between the groups.

478 patients were evaluated for long-term cardiac events. Baseline demographic, clinical, laboratory, angiographic data, scores, and medications given at discharge of those 478 patients are presented in Table 3. Age, IRA RCA ratio, stent length, CRP, peak troponin levels, GS, and furosemide use were higher in the RSS ≥ 7 group than the other groups; on the other hand, IRA-LAD ratio, CrCL, and LVEF were lower. The rates of hypertensive and diabetic patients, number of stents used, and HbA1c level were higher in the RSS ≥ 7 group than the RSS = 0 group. The rate of two-vessel disease was higher in the RSS < 7 groups than the others. Clopidogrel usage was low and ticagrelor usage was high in RSS = 0 group. The single vessel and three-vessel disease rates and SS and RSS levels were significantly different between the groups.

**Figure 3:**
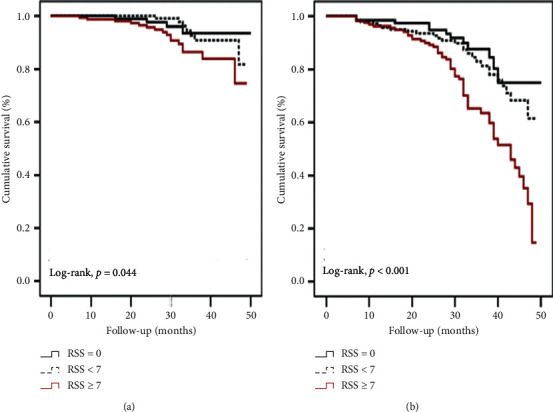
Kaplan–Meier survival curve for cardiovascular death (a) and MACE (b) (MACE: major adverse cardiac events).

In-hospital mortality, cumulative MACE, and subgroups of MACE were higher in the RSS ≥ 7 group than the other groups. One of the common safety endpoints was CIN and the common safety endpoint was more in the RSS ≥ 7 group than the other groups. Long-term clinical events were investigated and cardiac death, MACE, and recurrent revascularization rates were higher in RSS ≥ 7 than the other groups. In RSS ≥ 7 group, reinfarction rate was higher than RSS = 0.  Table 4 shows the ratio of in-hospital and long-term clinical events. Patients with ≥7 RSS score showed higher cardiac mortality and MACE rates compared with the other two groups with Kaplan–Meier analysis (*p*=0.044, *p* < 0.001, respectively). The Kaplan–Meier survival curves stratified according to RSS groups are presented in Figures 3(a) and 3(b).

Regardless of endpoints, all-cause mortality occurred in 35 patients. All-cause mortality occurred in 6 patients (4.6%) in the RSS = 0 group, 10 patients (5.5%) in the RSS < 7 group, and 19 (11.7%) patients in the RSS ≥ 7 group, and it was higher in the RSS ≥ 7 group than the other groups (*p*=0.031).

Univariate and multivariate Cox proportional hazards model was performed to define the factors independently influencing cardiac death and MACE and the risk factors are shown in Tables 5 and 6. On a multivariate model for SS, independent predictors for cardiac death were age and SS. On a multivariate model for RSS, independent predictors of cardiac death were age and RSS. On a multivariate model for SS, an independent predictor for MACE was SS. On a multivariate model for RSS, an independent predictor for MACE was RSS.

ROC analysis was performed for cutoff values of RSS, SS values, and GS, which predicted in-hospital mortality and MACE. The cutoff values for in-hospital mortality were found to be 10, 18.8, and 136, respectively. For in-hospital mortality, AUC of RSS was similar to GS but higher than the SS. Cutoff values for MACE were 6, 19, and 121, respectively. AUC of RSS was higher than AUC of SS and GS.

ROC analysis was performed for cutoff values of RSS, SS, and GS which predict long-term cardiac death and MACE. Cutoff values for cardiac death were found as 13, 22, and 126, respectively. AUC of RSS was higher than AUC of the SS and GS. Cutoff values for MACE were found as 11.5, 17, and 119, respectively. AUC of RSS was higher than the SS and GS.

**Figure 4:**
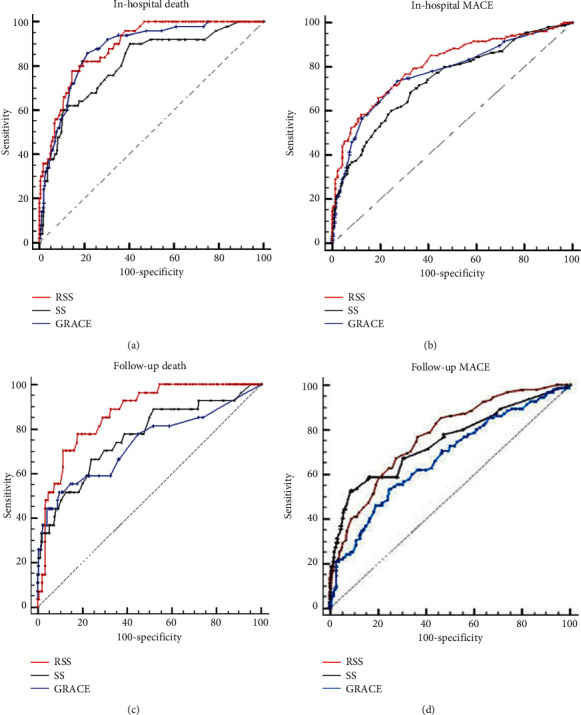
ROC curve analyses for endpoints.

Comparative results of ROC analysis related to in-hospital and follow-up clinical endpoints are presented in Table 7. All ROC analyses are shown in Figure 4.

## 4. Discussion

The main findings of our study are as follows. (1) Rates of in-hospital death, MACE, safety endpoint, and CIN were higher in the RSS ≥ 7 group. (2) In the RSS ≥ 7 group, long-term cardiac death and MACE were more than the other groups. During the study follow-up, the time spent without cardiac death and MACE was shorter in the RSS ≥ 7 group than the other groups. (3) When evaluated together with other risk factors, SS, RSS, and age were independent predictors of death during follow-up. SS and RSS were found as independent determinants for MACE in follow-up. (4) Predictive value of RSS was higher than SS for in-hospital death and higher than SS and GS for MACE. Predictive value of RRS was higher than SS and GS for long-term cardiac death and MACE.

Patients with STEMI are susceptible to significant morbidity and mortality, even when an early revascularization strategy such as P-PCI is performed. Thus, an appropriate risk stratification after P-PCI is essential to estimate the short- and long-term prognosis of these patients. Risk stratification in patients with STEMI is a major feature in real clinical practice, as it provides a broad perspective of patient outcomes based on patient characteristics and optimal treatment. The GS is considered the most robust score for evaluating the risk of patients with STEMI at the initial presentation. In addition to clinical risk assessment, angiographic characteristics that have additional independent prognostic significance include IRA, patency of the IRA, presence of multivessel disease, and lesion characteristics [25, 26]. In our study, RSS and GS showed similar efficacy for determining in-hospital mortality and in RSS ≥ 7 group, GS was higher than the other groups. Also, the ROC analysis for GS showed that AUC for in-hospital events was higher than AUC for the long-time period. In COX regression analysis, no correlation was found between GS and clinical events developed in long-time period. In our study, role of GS for predicting in-hospital events in STEMI patients treated with P-PCI was more significant than follow-up cardiac events. Since the GS is not based on severity of coronary artery disease, based on clinical findings at admission, it may be poor for predicting ischemic burden after P-PCI and clinical events in chronic phase.

In patients with STEMI, severity of coronary artery disease and extent of myocardial tissue under threat of ischemia are predictors of adverse cardiac events that may develop after P-PCI in hospital and follow-up period [27]. Therefore, presence and extent of additional coronary lesions are important for detection of myocardial tissue under threat of ischemia [28]. Reducing ischemic burden of patient would undoubtedly improve long-term prognosis, as exemplified by previous studies associating a short- and long-term survival benefit in patients with moderate to large amounts of inducible ischemia who were revascularized [5]. To address the question of the intervention of nonculprit vessel PCI in those with STEMI patients, scoring systems already used in clinical practice may be utilized. It is well accepted that amount of myocardium at risk at baseline and complexity and extent of CAD are directly correlated with occurrence of adverse events after revascularization [29].

SS has been recently developed as a combination of several previously validated angiographic classifications aiming to grade coronary anatomy concerning number of lesions and their functional impact, location, and complexity [30]. SS has proved to be useful in risk stratification of NON-STEMI and STEMI patients who underwent urgent PCI as an independent predictor of mortality, MACE, and stent thrombosis up to one-year follow-up. In patients undergoing P-PCI for STEMI, quantification of the presence, severity, and complexity of coronary vessel disease by SS is a useful tool in determining short-term and long-term outcomes independently of any other clinical, angiographic, and procedural characteristics. Patients with higher SS are at high risk and may need more intensive supportive and interventional management to improve their event-free survival [31].

CR seems to be a legitimate aim for patients with STEMI patients treated by PCI, whenever it can be safely achieved. In current practice, achieving CR is not always possible, and whether it is necessary for favorable outcomes is still a matter of debate. For patients presenting with NON-STEMI acute coronary syndromes, about 40% of patients have MVD and they are treated by PCI [32]. Indeed, whereas ICR may be unavoidable and detrimental in patients with complex coronary artery disease, in other cases (e.g., low CAD burden) the long-term prognosis may be reasonable with optimal medical therapy [8]. However, little is known about whether the specific vessels that are incompletely revascularized or degree of stenosis in those vessels are associated with different mortality rates [33]. Therefore, determining a reasonable level of revascularization in patients with STEMI undergoing P-PCI is of great importance. Number of untreated or unsuccessfully treated vessels was considered an indicator of ICR in many other studies. The concept of reasonable ICR has been proposed. Therefore, it is very important to determine a reasonable level of revascularization [13, 34].

Recently, the RSS measured after PCI emerged as an independent predictor of long-term clinical outcomes and tools for quantification of after validation by the preexisting clinical trials. The RSS is defined as SS, remaining after completion of PCI, including cases of staged PCI procedure [35, 36]. The RSS should be a more accurate marker of residual lesion and residual ischemic burden. High RSS was associated with greater incidence of MACE and conversely, low RSS was linked with a better outcome. Reasonable ICR can be associated with favorable long-term results after PCI. Several observational studies with PCI patients with or without acute coronary syndrome using either cutoff <8 or <5 for RSS found a significant reduction in MACE, death/MI/stroke, and unplanned revascularization procedures when the RSS after PCI was low. Therefore, reasonable ICR as determined by RSS carries better long-term prognosis in terms of clinical outcome than more extensive residual CAD in patients with MVD treated by PCI. RSS may improve the allocation of coronary disease for optimal mode of revascularization [13, 36].

Our study showed a relationship between RSS and adverse cardiac events during in-hospital and follow-up and its prognostic significance and role in post-revascularization risk classification in STEMI patients. We found that patients with greater residual coronary lesions and RSS ≥ 7 after PCI had higher clinical risks such as older age, lower LVEF, more comorbidities, bad clinical markers, negative procedural characteristics, and high SS and GS. These associations reflected a clinical phenomenon that patients at higher risks tend to receive ICR just as prior studies indicated [37]. In our study, RSS ≥ 7 group was associated with higher cardiac death and ischemic adverse events that developed in hospital and the long term. We compared the role of RSS in risk classification with risk parameters with proven efficacy such as GS and SS. In general, RSS was found to be more effective for predicting in-hospital and follow-up clinical events than the other two scores. While the GS and SS scores were associated with ischemia at the time of admission before revascularization, RSS showed the ischemia level associated with residual lesions after PCI. Degree of ischemia, induced by residual coronary lesions after PCI, is associated with early and late adverse cardiac events. This relationship may explain why predictive value of RSS is higher than the other two parameters.

Although no randomized controlled trials are investigating the relationship between RSS and clinical outcomes in the STEMI patient populations, some observational studies have investigated this association. In the study performed by Singbal, relationship between SS and RSS with 1-year mortality was investigated in patients undergoing P-PCI. Median SS value of the study population was 14 (IQR = 8–19.5) and RSS value was 2 (IQR = 2–7). Although SS and RSS were found to be associated with 1-year mortality, it was highly correlated with RSS. RSS > 12 predicted 1-year mortality with 90% specificity [38]. No different evaluation was made for in-hospital and follow-up deaths. In a previous study, Khan investigated the relationship between RSS and in-hospital clinical events in STEMI patients. In the study group, mean SS was 14.1 ± 8.2 and mean RSS was 4.7 ± 7.2. RSS > 8 was shown to be associated with adverse cardiac events [39]. In a study investigating the relationship between RSS, calculated after P-PCI, and clinical events developing in-hospital and follow-up, RSS > 8 was found to be associated with cardiac adverse events developing in hospital and in follow-up. Mean RSS = 4.2 ± 5.4 and median RSS = 2 were found in the study. Study showed that RSS was also an independent predictor of MACE and all-cause mortality during follow-up (OR 2.9 for RSS < 8 and 3.9 for RSS > 8), adding prognostic value over control variables and GS [40]. In a study by Burgess et al., cardiac death and MI were more common in RSS >8 group. RSS was found as an independent predictor of cardiac death, MI, unplanned revascularization, and MACE [41]. We evaluate our results together with current studies; RSS can be used as a reasonable parameter in prognostic evaluation and additional PCI decision after P-PCI. RSS ≥ 7-8 is associated with undesirable clinical events after P-PCI. Consideration of other clinical risk factors when performing additional PCI interventions in these patients may provide prognostic improvement.

## 5. Limitations

The main limitation of our study was undoubtedly its retrospective nonrandomized design. This was a retrospective observational and nonrandomized study conducted at a single hospital and as such has the inherent limitations and bias retrospective single-center studies. Although the multivariable analysis was performed for significant confounders, we cannot exclude the fact that other potential unmeasured confounders may affect the results. This study did not compare the prognostic impact of RSS with newer standardized SYNTAX-II (SS-II) scores that combine angiographic and clinical variables. The addition of these clinical variables to RSS may allow for better prediction of long-term outcomes when compared to the angiographic analysis of residual disease alone. On the other hand, it has been shown that the original SS and SS-II have similar efficacy [42]. We did not measure FFR systematically before and after PCI. Although FFR-guided PCI improved clinical outcomes compared with angiography guided PCI, many PCI procedures are still performed based on coronary angiographic findings. In our study, patients with MVD were not compared separately with or without additional PCI. We did not compare the difference between patients who underwent non-IRA PCI in the same procedure and those in the staged procedure. In addition, leaving the treatment method used to the operator's preference may have affected the results. However, medical treatment applied to patients and patient's compliance with treatment and lifestyle changes are important parameters that affect clinical results.

## 6. Conclusion

The present study shows that the RSS, in patients with STEMI undergoing P-PCI, provides important prognostic information with regard to in-hospital and follow-up clinical outcomes, including cardiac mortality and MACE. The RSS is an easy score to calculate the index of residual coronary disease severity and is intuitively more meaningful to apply in this setting. The RSS may improve the determination of an objective level of reasonable revascularization. RSS may help determine the level of revascularization, required to prevent ischemic cardiac events and improve prognosis after P-PCI; however, it can be used to guide additional PCI decisions.

## Figures and Tables

**Table 1:** In-hospital demographic, clinical, and laboratory characteristics and Grace Scores of patients.

Parameters	All patients (*n* : 538)	RSS = 0 (*n* : 131)	RSS < 7 (*n*: 188)	RSS ≥ 7 (*n* : 219)	*p* value
*Demographic parameters*					
Age (year)	61.9 ± 12.8	57.9 ± 12.4^*a*^	60.8 ± 12.3^*a*^	65.3 ± 12.7^*b*^	**<0.001**
Male (*n*%)	422 (78.4)	100 (76.3)	149 (79.3)	173 (79)	0.796

*Clinical history*					
HT (*n*%)	330 (61.3)	67 (51.1)^*a*^	111 (59)^*a*,*b*^	152 (69.4)^*b*^	**0.002**
DM (*n*%)	255 (47.4)	46 (35.1)^*a*^	85 (45.2)^*a*,*b*^	124 (56.6)^*b*^	**<0.001**
HL (*n*%)	326 (60.6)	72 (55)	115 (61.2)	139 (63.5)	0.283
CAD (*n*%)	122 (22.7)	23 (17.6)^*a*^	37 (19.7)^*a*^	62 (28.3)^*b*^	**0.032**
Smoking (*n*%)	217 (40.3)	55 (42)	72 (38.3)	90 (41.1)	0.769

*Clinical parameters*					
Anterior MI (*n*%)	256 (47.6)	64 (48.9)	97 (51.6)	95 (43.4)	0.240
Inferior MI (*n*%)	257 (47.8)	60 (45.8)	80 (42.6)	117 (53.4)	0.080
Other localization MI (*n*%)	25 (4.6)	7 (5.3)	11 (5.9)	7 (3.2)	0.407
SBP mm·Hg	131.3 ± 28.2	135.8 ± 23.3^*a*^	137.5 ± 24.1^*a*^	123.2 ± 32^*b*^	**<0.001**
DBP mm·Hg	77.6 ± 16.4	79.4 ± 13.7^*a*^	81.5 ± 15.1^*a*^	73 ± 17.8^*b*^	**<0.001**
Heart rate/min	82.5 ± 18.8	80.5 ± 16	81.2 ± 14.8	84.7 ± 22.9	0.065
Killip > 2 HF (*n*%)	102 (19)	8 (6.1)^*a*^	15 (8)^*a*^	79 (36.1)^*b*^	<0.001
LVEF (%)	55 (45–60)	60 (54–65)^*a*^	60 (50–60.5)^*a*^	50 (38–60)^*b*^	**<0.001**
Inotropic treatment (*n*%)	105 (19.5)	6 (4.6)^*a*^	14 (7.4)^*a*^	85 (38.8)^*b*^	**<0.001**
IV diuretic treatment (*n*%)	79 (14.7)	7 (5.3)^*a*^	18 (9.6)^*a*^	54 (24.7)^*b*^	**<0.001**
Gp2b3a treatment (*n*%)	86 (16)	15 (11.5)	31 (16.5)	40 (18.3)	0.236
IABP (*n*%)	23 (4.3)	0 (0)^*a*^	2 (1.1)^*a*^	21 (9.6)^*b*^	**<0.001**
Grace Score	107 (87–130)	95 (80–109)^*a*^	100 (81.5–122.5)^*a*^	124 (106–146)^*b*^	**<0.001**
Length of hospitalization (days)	5 (4–5)	4 (4-5)^*a*^	4 (4-5)^*a*^	5 (4–6)^*b*^	**0.008**

*Laboratory parameters*					
ABG level (mg/dl)	141 (115–197)	129 (105–187)^*a*^	138 (117–188)^*a*^	152 (124–225)^*b*^	**<0.001**
FBG level (mg/dl)	108.5 (92–152)	98 (85–142)^*a*^	107.5 (91–139)^*a*^	118 (97–165)^*b*^	**<0.001**
Hba1c	6.1 (5.6–7.3)	6 (5.5–6.9)^*a*^	6 (5.6–6.85)^*a*^	6.3 (5.7–8.1)^*b*^	**0.003**
A-Cre level (mg/dl)	0.89 (0.76–1.03)	0.86 (0.74–0.98)^*a*^	0.87 (0.75–1)^*a*^	0.92 (0.8–1.13)^*b*^	**<0.001**
PP-Cre level (mg/dl)	0.91 (0.8–1.11)	0.89 (0.79–1.02)^*a*^	0.9 (0.79–1.03)^*a*^	1 (0.8–1.42)^*b*^	**<0.001**
CreCl (ml/min)	87.26 (69.09–99.01)	92.36 (81–102)^*a*^	90.9 (78.38–101)^*a*^	80 (63.15–93.96)^*b*^	**<0.001**
Hb (g/dl)	13.94 ± 1.95	14.11 ± 1.8	14.1 ± 1.93	13.7 ± 2.02	0.065
WBC × 10^3^	10.94 (8.63–13.5)	10.41 (8.02–13.04)^*a*^	10.89 (8.37–12.8)^*a*^	11.3 (9.2–14.45)^*b*^	**0.003**
Neutrophil × 10^3^	7.17 (5–9.8)	6.39 (4.6–8.68)^*a*^	6.85 (5–9.63)^*a*,*b*^	7.5 (5.73–10.44)^*b*^	**0.006**
PLT × 10^3^	254 (208–305)	262 (213–308)	248 (207–294.5)	253 (205–310)	0.493
CRP (mg/dl)	1.79 (0.6–6.58)	1.39 (0.5–4.2)^*a*^	1.5 (0.57–5.12)^*a*^	2.7 (0.7–11.1)^*b*^	**0.001**
Peak Trop-T (ng/ml)	50000 (15.9–50000)	34.16 (8.1–50000)^*a*^	50000 (13.7–50000)^*b*^	50000 (35–50000)^*c*^	**<0.001**
Peak Trop-T (ng/ml) > 50000 (*n*%)	283 (52.6)	50 (38.2)^*a*^	95 (50.5)^*b*^	138 (63)^*c*^	**<0.001**

Data are presented as mean ± SD, median (0.250.75 percentiles), and *n* (%). ANOVA, Kruskal–Wallis test, and Pearson chi-square test were used and different letters show that there is a statistically significant difference. ABG: admission blood glucose, ACRE: admission creatinine, CAD: coronary artery disease, Cre: creatinine, CreCl: creatinine clearance, CRP: C-reactive protein, DBP: diastolic blood pressure, DM: diabetes mellitus, FBG: fasting blood glucose, Gp2b3a: glycoprotein 2b3a: antagonist, Hb: hemoglobin, HbA1C: hemoglobin A1C, HF: heart failure, HL: hyperlipidemia, HT: hypertension, IABP: intra-aortic balloon pump, IV: intravenous, LVEF: left ventricular ejection fraction, MI: myocardial infarction, PLT: platelet, PP-Cre: postprocedural creatinine, Trop: troponin, SBP: systolic blood pressure, and WBC: white blood cell.

**Table 2:** In-hospital angiographic and procedural data characteristics and SS/RS scores of patients.

Parameters	All patients	RSS = 0	RSS < 7	RSS ≥ 7	*p* value
One vessel (*n*%)	156 (29)	88 (67.2)^*a*^	51 (27.1)^*b*^	17 (7.8)^*c*^	**<0.001**
Multivessel disease (*n*%)	382 (71)	43 (32.8)^*a*^	137 (72.9)^*b*^	202 (92.2)^*c*^	**<0.001**
Three-vessel disease (*n*%)	164 (30.5)	6 (4.6)^*a*^	30 (16)^*b*^	128 (58.4)^*c*^	**<0.001**
Two-vessel disease (*n*%)	220 (40.9)	36 (27.5)^*a*^	109 (58)^*b*^	75 (34.2)^*a*^	**<0.001**

*Infarct related artery*					
LAD (*n*%)	247 (45.9)	67 (51.1)^*a*^	95 (50.5)^*a*^	85 (38.8)^*b*^	**0.023**
CX (*n*%)	95 (17.7)	25 (19.1)	34 (18.1)	36 (16.4)	0.806
RCA (*n*%)	176 (32.7)	34 (26)^*a*^	55 (29.3)^*a*,*b*^	87 (39.7)^*b*^	**0.013**
LMCA (*n*%)	7 (1.3)	0 (0)	0 (0)	7 (3.2)	NA
Other vessels (*n*%)	13 (2.4)	5 (3.8)	3 (1.6)	5 (2.3)	NA

*TIMI flow*					
PrePro TIMI 0 (*n*%)	296 (55)	59 (45)^*a*^	94 (50)^*a*^	143 (65.3)^*b*^	**<0.001**
PrePro TMI 1 (*n*%)	26 (4.8)	6 (4.6)	9 (4.8)	11 (5)	0.982
PrePro TIMI 2 (*n*%)	38 (7.1)	10 (7.6)	16 (8.5)	12 (5.5)	0.472
PrePro TIMI 3 (*n*%)	178 (33.1)	56 (42.7)^*a*^	69 (36.7)^*a*^	53 (24.2)^*b*^	**0.001**
PostPro TIMI 0 (*n*%)	11 (2)	0 (0)	0 (0)	11 (5)	NA
PostPro TMI 1 (*n*%)	16 (3)	0 (0)^*a*^	3 (1.6)^*a*,*b*^	13 (5.9)^*b*^	**0.003**
PostPro TIMI 2 (*n*%)	35 (6.5)	5 (3.8)^*a*^	8 (4.3)^*a*^	22 (10)^*b*^	**0.022**
PostPro TIMI 3 (*n*%)	476 (88.5)	126 (96.2)^*a*^	177 (94.1)^*a*^	173 (79)^*b*^	**<0.001**

*Type of stent*					
BMS (*n*%)	137 (25.5)	34 (26)	46 (24.5)	57 (26)	0.927
DES (*n*%)	401 (74.5)	97 (74)	142 (75.5)	162 (74)	0.927
Number of stents	1 (1–1)	1 (1–1)	1 (1–1)	1 (1–1)	0.067
1 (*n*%)	429 (79.7)	112 (85.5)	152 (80.9)	165 (75.3)	0.065
>1 (*n*%)	109 (20.3)	19 (14.5)	36 (19.1)	54 (24.7)	0.065
Stent length (mm)	22 (16–30)	20 (15–27)^*a*^	22.5 (17–30)^*b*^	24 (18–33)^*b*^	**0.004**
Stent diameter (mm)	3 (2.75–3)	3 (2.75–3.5)	3 (2.75–3)	3 (2.75–3)	0.258
Multivessel PCI (*n*%)	122 (22.7)	37 (28.2)^*a*^	54 (28.7)^*a*^	31 (14.2)^*b*^	**<0.001**
Different time (*n*%)	66 (54, 1)	22 (59, 5)^*a*^	34 (63)^*a*^	10 (32, 3)^*b*^	**0.017**
LAD (*n*%)	48 (39, 3)	14 (37, 8)	22 (40, 7)	12 (38, 7)	0.959
CX (*n*%)	51 (41, 8)	18 (48, 6)	21 (38, 9)	12 (38, 7)	0.600
RCA (*n*%)	40 (32, 8)	11 (29, 7)	20 (37)	9 (29)	0.671
SS	17 (10.5–24.5)	9 (7–14.5)^*a*^	15 (10–20.5)^*b*^	24.5 (19–31.5)^*c*^	**<0.001**
RSS	5 (2–11.5)	0 (0–0)^*a*^	3.8 (2–5)^*b*^	12.5 (10–19)^*c*^	**<0.001**

Data are presented as mean ± SD, median (0.250.75 percentiles), and *n* (%). ANOVA, Kruskal–Wallis test, and Pearson chi-square test were used and different letters show that there is a statistically significant difference. BMS: bare metal stent, CX: circumflex, DES: drug eluting stent, LAD: left anterior descending, LMCA: left main coronary artery, PCI: percutaneous coronary intervention, NA: not applicable, PostPro: postprocedural, PrePro: preprocedural, RCA: right coronary artery, RSS: residual SYNTAX score, SS: SYNTAX score, and TIMI: the thrombolysis in myocardial infarction.

**Table 3:** Long-term demographic, clinical, laboratory, and procedural characteristics, medication, and scores of patients.

Parameters	All patients (*n* : 478)	RSS = 0 (*n* : 130)	RSS < 7 (*n* : 185)	≥7 (*n* : 163)	*p* value
*Demographic parameters*					
Age (year)	62 ± 13	58 ± 12^*a*^	61 ± 12^*a*^	65 ± 13^*b*^	**<0.001**
Male gender (*n*%)	371 (77.6)	101 (77.7)	146 (78.9)	124 (76.1)	0.817

*Clinical history*					
HT (*n*%)	285 (59.6)	67 (51.5)^*a*^	109 (58.9)^*a*,*b*^	109 (66.9)^*b*^	**0.028**
DM (*n*%)	216 (45.2)	45 (34.6)^*a*^	84 (45.4)^*a*,*b*^	87 (53.4)^b^	**0.006**
HL (*n*%)	282 (59)	72 (55.4)	114 (61.6)	96 (58.9)	0.541
CAD (*n*%)	104 (21.8)	22 (16.9)	37 (20)	45 (27.6)	0.067
Smoking (*n*%)	186 (38.9)	55 (42.3)	70 (37.8)	61 (37.4)	0.646

*Laboratory parameters*					
CreCl (ml/min)	89.05 (72.6–100)	92.32 (82–101)^*a*^	91 (79–101.03)^*a*^	83 (67–95.8)^*b*^	**<0.001**
HbA1c	6 (5.6–7)	5.9 (5.5–6.8)^*a*^	6 (5.6–6.8)^*a*,*b*^	6.2 (5.7–7.4)^*b*^	0.049
Hb (g/dl)	14.17 ± 4.92	14.05 ± 1.98	14.13 ± 1.91	14.31 ± 8	0.899
WBC × 10^3^	10.73 (8.43–13)	10.04 (8–12.78)	10.87 (8.25–12.73)	10.9 (8.74–13.39)	0.157
PLT × 10^3^	253.5 (208–304)	262 (213–306)	247 (207–292)	247 (205–308)	0.397
CRP (mg/dl)	1.8 (0.61–6)	1.38 (0.5–3.58)^*a*^	1.5 (0.57–4.93)^*a*^	3.15 (0.78–11.1)^*b*^	**<0.001**
Peak Trop-T (ng/ml)	50000 (14.59–50000)	34.37 (8.4–50000)^*a*^	48.66 (13.3–50000)^*a*^	50000 (31.9–50000)^*b*^	**<0.001**
>50000 (*n*%)	244 (51)	50 (38.5)^*a*^	91 (49.2)^*a*^	103 (63.2)^*b*^	**<0.001**
LVEF	58 (50–60)	60 (55–65)^*a*^	60 (50–61)^*a*^	52 (45–60)^*b*^	**<0.001**
Grace Score	105 (85–124)	94.5 (80–109)^*a*^	99 (81–121)^*a*^	120 (103–138)^*b*^	**<0.001**

*Angiographic characteristic*					
One vessel (*n*%)	149 (31.2)	87 (66.9)^*a*^	51 (27.6)^*b*^	11 (6.7)^*c*^	**<0.001**
Two vessels (*n*%)	202 (42.3)	35 (26.9)^*a*^	105 (56.8)^*b*^	62 (38)^*a*^	**<0.001**
Three vessels (*n*%)	149 (31.2)	7 (5.4)^*a*^	30 (16.2)^*b*^	91 (55.8)^*c*^	**<0.001**
IRA-LAD (*n*%)	217 (45.4)	65 (50)^*a*^	95 (51.4)^*a*^	57 (35)^*b*^	**0.004**
IRA-CX (*n*%)	90 (18.8)	25 (19.2)	34 (18.4)	31 (19)	0.979
IRA-RCA (*n*%)	158 (33.1)	35 (26.9)^*a*^	53 (28.6)^*a*^	70 (42.9)^*b*^	**0.004**
BMS (*n*%)	119 (24.9)	34 (26.2)	44 (23.8)	41 (25.2)	0888
DES (*n*%)	41 (25.2)	96 (73.8)	141 (76.2)	122 (74.8)	0888
Number of stents	1 (1-1)	1 (1-1)^*a*^	1 (1-1)^*a*,*b*^	1 (1-2)^*b*^	**0.021**
1 (*n*%)	380 (795)	112 (86.2)^*a*^	149 (80.5)^*a*,*b*^	119 (73)^*b*^	**0.020**
≥1 (*n*%)	98 (20.5)	18 (13.8)^*a*^	36 (19.5)^*a*,*b*^	44 (27)^*b*^	**0.020**
Stent length (mm)	22 (16–30)	19.5 (15–26)^*a*^	24 (17–30)^*a*^	24 (18–34)^*b*^	**0.002**
Stent diameter (mm)	3 (2.75–3)	3 (2.75–3.5)	3 (2.75–3)	3 (2.75–3)	0.262
SS	16 (10–22.5)	9.5 (7–15)^*a*^	15 (10–20)^*b*^	23.5 (18–29)^*c*^	**<0.001**
RSS	4 (0–8.5)	0 (0-0)^*a*^	3.5 (2–5)^*b*^	12 (8–15.5)^*c*^	**<0.001**

*Medication*					
Beta-blocker (*n*%)	448 (93.7)	123 (94.6)	174 (94.1)	151 (92.6)	0.765
ASA (*n*%)	476 (99.6)	129 (99.2)	185 (100)	162 (99.4)	NA
Clopidogrel (*n*%)	139 (29.1)	26 (20)^*a*^	61 (33)^*b*^	52 (31.9)^*b*^	**0.028**
Prasugrel (*n*%)	15 (3.1)	2 (1.5)	7 (3.8)	6 (3.7)	0.471
Ticagrelor (*n*%)	319 (66.7)	101 (77.7)^*a*^	115 (62.2)^*b*^	103 (63.2)^*b*^	**0.008**
ACEI-ARB (*n*%)	396 (82.8)	105 (80.8)	152 (82.2)	139 (85.3)	0.568
Statin (*n*%)	464 (97.1)	126 (96.9)	181 (97.8)	157 (96.3)	0.699
MRA (*n*%)	31 (6.5)	6 (4.6)	10 (5.4)	15 (9.2)	0.213
Furosemide (*n*%)	50 (10.5)	9 (6.9)^*a*^	12 (6.5)^*a*^	29 (17.8)^*b*^	**0.001**
Follow-up (months)	29 (18–35)	28 (12–34)	28 (14–38)	30 (23–34)	0.055

Data are presented as mean ± SD, median (0.250.75 percentiles), and *n* (%). ANOVA, Kruskal–Wallis test, and Pearson chi-square test were used and different letters show that there is a statistically significant difference. ACEI-ARB: angiotensin converting enzyme inhibitors-angiotensin receptor blockers, ASA: acetylsalicylic acid, BMS: bare metal stent, CAD: coronary artery disease, CreCl: creatinine clearance, CRP: C-reactive protein, CX: circumflex, DES: drug eluting stent, DM: diabetes mellitus, Hb: hemoglobin, HbA1C: hemoglobin A1C, HF: heart failure, HL: hyperlipidemia, HT: hypertension, IRA: infarct related artery, IV: intravenous, LAD: left anterior descending, LVEF: left ventricular ejection fraction, MRA: mineralocorticoid receptor antagonist, MI: myocardial infarction, NA: not applicable, PLT: platelet, RCA: right coronary artery, RSS: residual SYNTAX score, SS: SYNTAX score, Trop: troponin, and WBC: white blood cell.

**Table 4:** In-hospital and long-term adverse events according to RSS.

	All patients (*n* : 538)	RSS = 0 (*n* : 131)	RSS < 7 (*n* : 188)	RSS ≥ 7 (*n*: 219)	*p* value
*In-hospital primary endpoint*					
Cardiac death	47 (8.7)	1 (0.8)^*a*^	2 (1.1)^*a*^	44 (20.1)^*b*^	**<0.001**

*In-hospital secondary endpoint*					
MACE	152 (28.3)	16 (12.2)^*a*^	25 (13.3)^*a*^	111 (50.7)^*b*^	**<0.001**
Reinfarction	33 (6.1)	2 (1.5)^*a*^	2 (1.1)^*a*^	29 (13.2)^*b*^	**<0.001**
DHF	107 (19.9)	6 (4.6)^*a*^	19 (10.1)^*a*^	82 (37.4)^*b*^	**<0.001**
VT/VF	70 (13)	10 (7.6)^*a*^	10 (5.3)^*a*^	50 (22.8)^*b*^	**<0.001**
In-hospital safety endpoints	96 (17.8)	14 (10.7)^*a*^	19 (10.1)^*a*^	63 (28.8)^*b*^	**<0.001**
CIN	86 (16)	12 (9.2)^*a*^	17 (9)^*a*^	57 (26)^*b*^	**<0.001**
Bleeding	15 (2.8)	2 (1.5)	4 (2.1)	9 (4.1)	0.289

	All patients (*n*: 478)	RSS = 0 (*n*: 130)	0 < RSS < 7 (*n* : 185)	RSS ≥ 7 (*n*: 163)	*p* value
*Long-term primary endpoint*					
Cardiac death	27 (5.6)	4 (3.1)^*a*^	7 (3.8)^*a*^	16 (9.8)^*b*^	**0.017**

*Long-term secondary endpoint*					
MACE	102 (20.5)	14 (9.2)^*a*^	29 (14.6)^*a*^	59 (35.6)^*b*^	**<0.001**
Reinfraction	32 (6.7)	4 (3.1)^*a*^	10 (5.4)^*a*^	18 (11.1)^*b*^	**0.032**
Heart failure	15 (3.1)	2 (1.5)	5 (2.7)	8 (4.9)	0.236
Repeat revascularization	28 (5.9)	4 (3.1)^*a*^	7 (3.8)^*a*^	17 (10.4)^*b*^	**0.009**

Data are presented as *n* (%). Pearson chi-square test. Different lower case letters in a row indicate statistically significant difference between groups. CIN: contrast induced nephropathy, DHF: decompensated heart failure, MACE: major adverse cardiac events, RSS: residual SYNTAX score, and VT/VF: ventricular tachycardia/fibrillation.

**Table 5:** Univariate and multivariate cox regression analysis for determining the risk factors associated with long-term cardiac death.

Variables	Univariate analysis		Multivariate analysis for SS		Multivariate analysis for RSS	
	HR (95% CI)	*p*	HR (95% CI)	*p*	HR (95% CI)	*p*
Age	1.181 (1.13–1.234)	**<0.001**	1.156 (1.093–1.223)	<0.001	1.171 (1.104–1.243)	**<0.001**
Male gender	0.41 (0.19–0.885)	**0.023**	1.895 (0.753–4.766)	0.175	2.162 (0.858–5.448)	0.102
HT	0.697 (0.325–1.493)	0.353	—	—	—	—
DM	1.69 (0.784–3.643)	0.180	—	—	—	—
HL	0.583 (0.273–1.244)	0.163	—	—	—	—
CAD	0.998 (0.403–2.474)	0.997	—	—	—	—
Smoking	0.254 (0.096–0.674)	**0.006**	0.891 (0.255–3.117)	0.857	0.867 (0.259–2.905)	0.817
Hba1c	1.091 (0.875–1.362)	0.439	—	—	—	—
CreCl	0.975 (0.96–0.989)	**0.001**	1.008 (0.982–1.035)	0.550	1.013 (0.987–1.04)	0.335
Hb	0.795 (0.671–0.941)	**0.008**	0.955 (0.768–1.189)	0.682	0.972 (0.815–1.159)	0.751
LVEF	0.973 (0.94–1.008)	0.125	—	—	—	—
WBC	0.956 (0.855–1.068)	0.425	—	—	—	—
CRP	0.956 (0.855–1.068)	0.868	—	—	—	—
PLT	1.002 (0.997–1.006)	0.399	—	—	—	—
Stent diameter	0.747 (0.302–1.849)	0.528	—	—	—	—
Stent length	0.981 (0.946–1.017)	0.294	—	—	—	—
Number of stents	0.529 (0.203–1.378)	0.192	—	—	—	—
Type of stent			—	—	—	—
BMS	Ref	—	—	—	—	—
DES	0.753 (0.338–1.679)	0.488	—	—	—	—
Anterior infarction	0.775 (0.354–1.694)	0.522	—	—	—	—
Inferior infarction	1.203 (0.562–2.575)	0.634	—	—	—	—
Other infarction	1.257 (0.297–5.312)	0.756	—	—	—	—
SS	1.118 (1.077–1.161)	**<0.001**	1.074 (1.032–1.118)	**<0.001**	—	—
RSS	1.115 (1.076–1.157)	**<0.001**	—	—	1.083 (1.038–1.13)	**<0.001**
Grace Score	1.046 (1.032–1.061)	**<0.001**	1.019 (0.997–1.041)	0.085	1.017 (0.996–1.039)	0.113

BMS: bare metal stent, CAD: coronary artery disease, CreCl: creatinine clearance, CRP: C-reactive protein, DES: drug eluting stent, DM: diabetes mellitus, Hb: hemoglobin, HbA1C: hemoglobin A1C, HL: hyperlipidemia, HT: hypertension, LVEF: left ventricular ejection fraction, PLT: platelet, RSS: residual SYNTAX score, SS: SYNTAX score, and WBC: white blood cell.

**Table 6:** Univariate and multivariate cox regression analysis for determining the risk factors associated with long-term MACE.

Variables	Univariate analysis		Multivariate analysis for SS		Multivariate analysis for RSS	
	HR (95% CI)	*p*	HR (95% CI)	*p*	HR (95% CI)	*p*
Age	1.039 (1.021–1.057)	**<0.001**	1.006 (0.982–1.03)	0.619	1.012 (0.989–1.037)	0.309
Male gender	0.679 (0.438–1.053)	0.084	1.145 (0.692–1.894)	0.598	1.208 (0.728–2.005)	0.463
HT	1.101 (0.719–1.688)	0.657	—	—	—	—
DM	1.635 (1.088–2.458)	**0.018**	0.964 (0.56–1.659)	0.894	1.016 (0.59–1.749)	0.954
HL	0.986 (0.647–1.501)	0.946	—	—	—	—
KAH	1.308 (0.833–2.054)	0.244	—	—	—	—
Smoking	0.554 (0.36–0.853)	**0.007**	0.68 (0.413–1.118)	0.128	0.712 (0.436–1.162)	0.174
Hba1c	1.155 (1.035–1.288)	**0.010**	1.112 (0.95–1.303)	0.186	1.106 (0.943–1.296)	0.214
CreCl	0.988 (0.98–0.997)	**0.008**	1.004 (0.992–1.016)	0.543	1.009 (0.997–1.021)	0.164
Hb	0.973 (0.889–1.065)	0.550	—	—	—	—
LVEF	0.965 (0.949–0.983)	**<0.001**	0.999 (0.978–1.02)	0.898	0.99 (0.97–1.011)	0.367
WBC	0.968 (0.912–1.027)	0.275	—	—	—	—
CRP	0.999 (0.997–1.001)	0.116	—	—	—	—
PLT	0.996 (0.994–1.004)	0.117	—	—	—	—
Stent diameter	0.605 (0.365–1.001)	0.052	0.823 (0.481–1.409)	0.478	0.859 (0.503–1.467)	0.577
Stent length	1.002 (0.986–1.019)	0.764	—	—	—	—
Number of stents	0.997 (0.705–1.41)	0.986	—	—	—	—
Type of stent			—	—	—	—
BMS	Ref	—	—	—	—	—
DES	0.997 (0.705–1.41)	0.477	—	—	—	—
Anterior infarction	1.311 (0.876–1.961)	0.188	—	—	—	—
Inferior infarction	0.741 (0.493–1.113)	0.148	—	—	—	—
Other infarction	1.114 (0.487–2.549)	0.799	—	—	—	—
SS	1.088 (1.067–1.111)	**<0.001**	1.076 (1.051–1.102)	**<0.001**	—	—
RSS	1.083 (1.061–1.105)	**<0.001**	—	—	1.07 (1.043–1.097)	**<0.001**
Grace score	1.019 (1.013–1.026)	**<0.001**	1.01 (0.999–1.021)	0.066	1.01 (0.999–1.021)	0.078

BMS: bare metal stent, CAD: coronary artery disease, CreCl: creatinine clearance, CRP: C-reactive protein, DES: drug eluting stent, DM: diabetes mellitus, Hb: hemoglobin, HbA1C: hemoglobin A1C, HL: hyperlipidemia, HT: hypertension, LVEF: left ventricular ejection fraction, MACE: major adverse cardiac events, PLT: platelet, RSS: residual SYNTAX score, SS: SYNTAX score, WBC: white blood cell.

**Table 7:** Cutoff values and comparison results of RSS, SS, and Grace Scores related to in-hospital and follow-up primary and secondary endpoints.

	AUC	Cutoff Value	*p*	Sensitivity (%)	Specificity (%)	RSS versus SS	RSS versus Grace S.
						*p*	*p*

*In-hospital mortality*						0.035	0.651
RSS	0.889	>10	<0.001	86	78.69		
SS	0.809	>18.8	<0.001	90	59.43		
Grace Score	0.876	>136	<0.001	78	85.25		
*In-hospital MACE*						0.025	0.041
RSS	0.806	>6	<0.001	73.55	72.58		
SS	0.734	>19	<0.001	68.39	67.36		
Grace Score	0.770	>121	<0.001	65.8	80.9		
*Follow-up mortality*						0.02	0.001
RSS	0.870	>13	<0.001	77.78	82.04		
SS	0.763	>22	<0.001	66.67	75.83		
Grace Score	0.733	>126	<0.001	51.85	90.02		
*Follow-up MACE*						0.03	0.004
RSS	0.819	11.5	<0.001	76.84	63.45		
SS	0.747	17	<0.001	52.63	91.38		
Grace Score	0.673	129	<0.001	53.68	75.2		

AUC: area under the curve, MACE: major adverse cardiac events, RSS: residual SYNTAX score, and SS: SYNTAX score.

## Data Availability

The data used to support the findings of this study are included within the article.
